# Improvement of the Adhesion and Diamond Content of Electrodeposited Cu/Microdiamond Composite Coatings by a Plated Cu Interlayer

**DOI:** 10.3390/ma14102571

**Published:** 2021-05-15

**Authors:** Xiaoli Wang, Chau-Chang Chou, Liberty Tse-Shu Wu, Rudder Wu, Jyh-Wei Lee, Horng-Yi Chang

**Affiliations:** 1 School of Mechanical Engineering, Jiangsu Ocean University, Lianyungang 222005, China; 2006000018@jou.edu.cn; 2 Jiangsu Key Laboratory of 3D Printing Equipment and Application Technology, Nantong Institute of Technology, Nantong 226007, China; 3 Marine Resources Development Institute of Jiangsu, Jiangsu Ocean University, Lianyungang 222005, China; 4 Department of Mechanical and Mechatronic Engineering, National Taiwan Ocean University, Keelung 20224, Taiwan; 5 Center for Marine Mechatronic Systems, National Taiwan Ocean University, Keelung 20224, Taiwan; 6 Department of Metallurgy, Graduate School of Engineering, Tohoku University, Sendai 980-8576, Japan; wu.liberty.tse.shu.a1@tohoku.ac.jp; 7 Research Center for Structural Materials, National Institute for Materials Science, Tsukuba 305-0047, Japan; wu.rudder@nims.go.jp; 8 Department of Materials Engineering, Ming Chi University of Technology, New Taipei 243303, Taiwan; jefflee@mail.mcut.edu.tw; 9 Center for Plasma and Thin Film Technology, Ming Chi University of Technology, New Taipei 243303, Taiwan; 10 Department of Mechanical Engineering, Chang Gung University, Taoyuan 333323, Taiwan; 11 Plastic and Reconstructive Surgery, and Craniofacial Research Center, Chang Gung Memorial Hospital, Taoyuan 333424, Taiwan; 12 Department of Marine Engineering, National Taiwan Ocean University, Keelung 20224, Taiwan; hychang@mail.ntou.edu.tw

**Keywords:** composite electrodeposition, copper interlayer, micro-diamond particle, uniformity, adhesion, brass

## Abstract

Diamond-incorporated copper metal matrix layers were fabricated on brass substrates by using electrodeposition technology in this study. To improve the adhesion of the composite coatings on the brass substrate, a plated copper was applied as the interlayer between the multilayers and the substrate. The surface morphologies of the interlayer and the diamond-incorporated copper composite layers were studied by scanning electron microscopy. The effect of the copper interlayer on the incorporation and the distribution of the diamond content in the coatings was analyzed by surface roughness, electrochemical impedance spectroscopy, and cyclic voltammetry. The diamond content of the composite coating was measured by energy-dispersive X-ray. The film thickness was evaluated by the cross-sectional technique of focused ion beam microscopy. The element, composition, and crystallization direction of diamond with Cu matrix was measured by X-ray diffraction and transmission electron microscope. The adhesion of the multilayers was studied by scratch tests. The experiment results indicated that the diamond content and distribution of the coating were higher and more uniform with the Cu interlayer than that without one. The plated copper interlayer reduced the electrical double-layer impedance and enhanced the adsorption of diamond particles by the surrounding Cu ions, which promoted the diamond content in the composite coatings. The roughened surface caused by the plated Cu interlayer also improved the substrate’s mechanical interlock with the composite coating, which contributed to the strong adhesion between them.

## 1. Introduction

Because of the rapid development of manufacturing industry, the demand for micro-machining has been increasing rapidly. The miniaturization of cutting tool has increased the demands for manufacturing precision. Electrical discharge machining is often used to manufacture various types of micro-parts [1], as well as micro-cutting tools (e.g., those for micro-drilling, micro-cutting, and micro-repair). As the substrate is supposed to be the micro-milling/electro-machining tool to cut brittle or fragile workpieces, the application of brass can thus result in a better toughness and is still excellent to absorb the impact from contact. However, the discharged debris accumulate and increase on ordinary brass electrodes with the increase of discharge wear loss during the process, which cannot be immediately expelled from the discharge gap by the working fluid. As a consequence, the probability of the secondary discharge increases, which accelerates the electrode loss and reduces the machining accuracy [2,3,4].

By depositing composite layers onto substrates, composite electrodeposition effectively strengthens the surface of electrodes [5,6,7]. Diamond possesses unique physical properties, including high hardness, high thermal conductivity [8,9], and low thermal expansion. It is a valuable particle reinforcement material [10]. To increase the wear resistance and corrosion resistance, as well as to improve the hardness of cutting tools, the surface of cutting tools is often coated with diamond composites [11,12,13]. Applying the composite coatings on an electrode for hybrid machining can not only increase the wear resistance and corrosion resistance of electrode surface, but can also simultaneously remove the debris and heat on the electrical discharge surface of the workpiece, thereby improving the machining quality with low tool wear rate. Hsue et al. [14] investigated the coating of cylindrical tungsten carbide drill bits, where they added micron-diamond particles into nickel–cobalt composite coatings, which were used as the electrode of electro discharge micro-drill machining; they reported a surface roughness of 0.107 μm by using 6–12 μm diamond particles. To improve the adhesion of the coating to the substrate, chemical etching methods were used to roughen the substrates’ surfaces as the pretreatment [9,15,16,17], which effectively improved the adhesion of the deposited layer. However, the process of the chemical etching was generally time-consuming and induced the problem of chemical pollution.

Some researchers prepared an interlayer before composite deposition to strengthen the adhesion between the coating and the substrate, which achieved good results [18,19,20]. Some works mentioned that plating copper before nickel–diamond composite electrodeposition helps to improve the adhesion of the film to the substrate. For example, Rajasekaran et al. [21] and Hattori et al. [22] prepared a film of Ni/Cu multilayers during the electrodeposition process and confirmed that it possesses very good resistance to corrosion, with its abrasion improved by one-fifth compared to the lack of a Cu-plated layer. In 2018, Shen et al. [23] used a rotating-jet electrodeposition technique to plate Cu–Ni multilayer films. There was a hybrid interlayer formed between the Cu layer and Ni layer and the actual structure of the multilayer film was Cu/CuNi/Ni. This special structure enhanced the interface of the nano-multilayer film and was a major factor to improve film’s performance. In 2014, Qiu et al. [24] used copper–diamond composite electrodeposition by conducting a plated pure copper in advance. They used hot-filament chemical vapor deposition (CVD) to deposit diamonds and thus to achieve a better surface structure performance; however, the temperature of CVD was higher than the melting point of copper, which caused copper to dissolve. The authors did not explore whether diamond particles can be incorporated with the deposited layer. The Cu/Ti interlayer can improve the adhesion force of the thin film as well as increase the contact area of the film/substrate boundary [25]. The adhesion, corrosion, and wear resistance of Ni electrodeposited coating by applying Cu interlayer were improved [26,27]. Since copper possesses larger surface energy than interfacial energy, a wetted diamond/metal interface forms during the diamond deposition process, providing a stronger adhesion force as compared with other growing modes [28]. The aforementioned studies on plated copper interlayer prior to the electrodeposition of nickel or copper helps to increase the adhesion between the nickel layer and the substrate. To improve the diamond content of the coating, some scholars studied the effects of process parameters in the composite electrodeposition. Malathy et al. [29] studied the pH value and the temperature of the electrolyte on the coating’s performance. Other authors [10,30,31,32] studied the effects of diamond concentration, stirring speed, and diamond’s particle size on the diamond content of the coating surface, and thereby analyzed the effects on mechanical properties such as hardness and wear resistance.

As an extensive study [4] has been carried out to investigate the adhesion, anti-corrosion, and discharge wear rate of the whole copper–diamond composite coating on a brass substrate, the existence of the Cu interlayer is critical but still unclear. In this study, to understand the essential effect of the Cu interlayer, the copper–diamond composite electrodeposition was implemented on a brass substrate in two situations, namely, those with a plated Cu interlayer and without one. Furthermore, a Cu outmost layer was applied instead of the Ni one in [4] to avoid the influence of Ni layer on the evaluation of the coating’s electrochemical and mechanical properties. The adhesion and the diamond content of the coatings, with and without Cu interlayer, were studied by surface morphology, structure composition, electrochemical behavior, and scratch test.

## 2. Materials and Methods

### 2.1. Electrodeposition of Multilayer Films on Brass Plate

**Figure 1:**
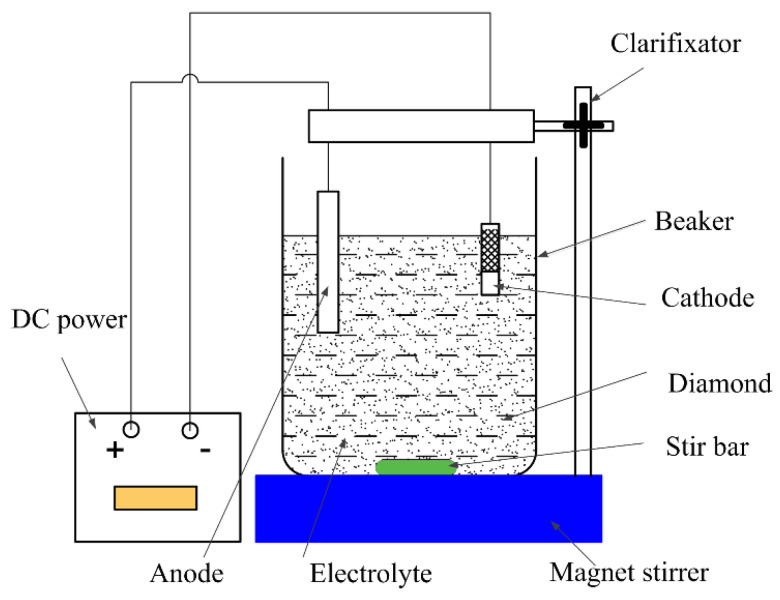
Principle diagram of copper–diamond composite electrodeposition.

A principle diagram of the device for the copper–diamond particle composite electrodeposition is shown in Figure 1. Diamond particles were uniformly suspended in the electrolyte by stirring with a stirrer. Direct current power was applied. The anode was a pure copper sheet electrode, and the cathode, a brass sheet electrode. In the pre-treatment process, specimen A and specimen B were ground by #400, #600, #800, and #1000 abrasive paper, respectively. Both specimens were placed in alcohol, subjected to ultrasonic vibration cleaning for 5 min, and then removed and blow-dried for use.

The dimensions of brass cathodes were 50 mm × 15 mm × 1 mm and sequentially ground by #400, #600, and # 800 abrasive paper in water, ultrasonically cleaned in 95 vol.% alcohol solution for 5 min, and dried in air. They were pasted with insulating tape to leave an exposed area of 12 mm × 15 mm for the deposition. The anode was a copper plate of 50 mm × 15 mm × 0.8 mm. The anode’s immersed area was 32 mm × 15 mm. The distance between the anode and cathode was 5 cm.

The electrodeposition process was divided into three steps. The first and second steps were the same as described in our previous work [4]. In brief, the first step was to plate the Cu interlayer on the brass substrate for 5 min in an electrolyte that contained 250 g/L CuSO_4_·5H_2_O and 0.5 M H_2_SO_4_ under the current density of 5 A/dm^2^ at 25 °C. The second step was a composite electrodeposition process under the current density of 5 A/dm^2^ with a stirring speed of 200 rpm at 25 °C. The composition of the electrolyte solution and parameters of composite electrodeposition are shown in Table 1. The particle size of diamond particle was in the range of 2–4 μm. The concentration of the diamond in the electrolyte was 10 g/L. The third step was the outmost Cu layer to fix the diamond particles protruded from the surface for 3 min under the current density of 3 A/dm^2^. The electrolyte was the same as the first step. Then the samples were ultrasonically cleaned in 95 vol.% alcohol solution for 5 min and blow-dried. For the comparative analysis of the effect of this stage, specimen A did not have a plated Cu interlayer, while specimen B had a plated Cu interlayer. To obtain the same thickness of the coatings, specimen A underwent an extra 5-min 2nd step, i.e., composite electrodeposition. The corresponding processing durations in each step are shown in Table 2.

### 2.2. Measurements

Scanning electron microscopy (SEM, Hitachi-4800, Hitachi Ltd., Tokyo, Japan) was used to observe the surface morphology of various specimens. Surface composition analysis of the coatings was conducted by energy-dispersive X-ray (EDX, Hitachi-4800, Hitachi Ltd., Tokyo, Japan). The roughness was measured by a roughness meter (KOSAKA LAC SC 500, standard JIS94, Kosaka Laboratory Ltd., Tokyo, Japan). The measurement range was 1.6 mm with sliding speed of 0.5 mm/s, and the stylus was diamond tipped with a radius of 2 μm and apex angle of 60°. The applied force was 0.75 mN. An electrochemical test with a three-electrode cell was conducted. A brass substrate with a Cu interlayer, a platinum electrode, and a saturated AgCl electrode were used as the working electrode, the auxiliary electrode, and the reference electrode, respectively. The experimental instrument was a CHI 6273 electrochemical workstation. The solution was 6.5 g/L potassium ferricyanide. Electrochemical impedance spectroscopies (EIS) were evaluated under the amplitude of 0.3 V and the frequency range of 100 kHz to 0.1 Hz. Cyclic voltammetry curves were performed for 5 cycles at a sweep rate of 0.05 V/s. The middle cycle was chosen as the typical one. The focused ion beam (FIB, Hitachi NB 5000, Hitachi Ltd., Tokyo, Japan) and the transmission electron microscope (TEM, JEOL JEM-2100, Tokyo, Japan) were used to study the interface. Energy dispersive spectroscopy (EDS) provided the elemental and chemical analysis of a sample inside the TEM. The scratch tester (Anton Paar RST3, Anton Parr GmbH, Graz, Austria) with a Rockwell diamond indenter type (serial no. AM-260) was adopted to linearly scratch the samples along a 5 mm path with progressive loading from 0.5 N to 20 N at a sliding speed of 10 mm/min. All specimens in this work were built with more than 3 samples. Each specimen was investigated by their surface roughness and surface morphology before the other measurements. The average and standard deviation statistic method was used for the surface roughness data and the weight percentages of element composition data. A two-sample t-test was conducted to verify the significant difference between two data sets.

## 3. Results and discussion

### 3.1. Roughness and Electrochemical Characteristics of Substrate with Cu Interlayer

**Figure 2:**
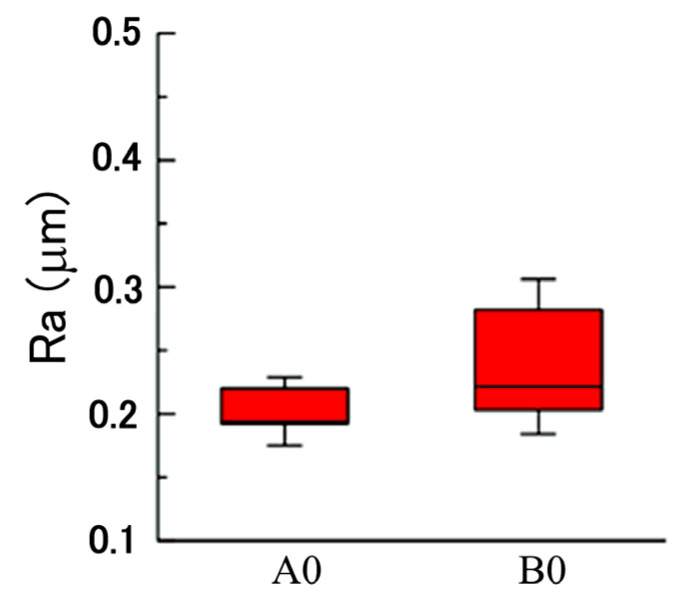
Comparison on roughness before (A0) and after (B0) a plated Cu interlayer on the brass substrate.

**Figure 3:**
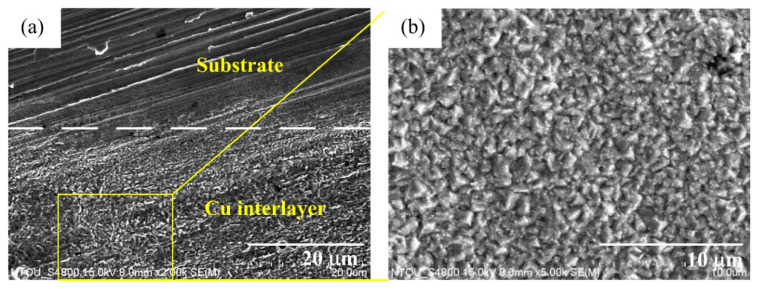
Microscopic morphology of the plated Cu interlayer. (**a**) the plated Cu interlayer partially on substrate (lower part), and (**b**) the magnified Cu interlayer.

We believe that a plated Cu interlayer before copper–diamond composite electrodeposition is extremely important. The main function of this process can be summarized into two points. First, a plated Cu interlayer generated a roughing effect on the surface. Using a roughness meter to measure the surface roughness before and after a plated Cu interlayer, nine roughness values of different positions from three specimens were measured, with the maximum value and minimum value removed before averaging to generate a box plot for surface roughness (center line average, Ra). A comparison can be seen in Figure 2, where A0 is the brass substrate before a plated Cu interlayer, B0 is the brass substrate after a plated Cu interlayer, and the error bar is the standard deviation. According to the roughness comparison in Figure 2, the increase in overall roughness on the surface after a plated Cu interlayer was not significant (*p* > 0.1). Nevertheless, the microscopic morphology of the plated Cu interlayer partially on substrate (lower part) in Figure 3 indicated a roughing tendency on the surface, since pits and bumps of a certain size were formed on the surface of the plated Cu interlayer, which provided more nucleation sites for the copper–diamond composite electrodeposition in the second stage. More nucleation sites facilitated the attachment of diamond particles, so that the composite coating and the matrix generated mutual embedment action through the pits and bumps. At the same time, the mutual embedment action enhanced the adhesion of composite coating between the plated Cu interlayer and substrate, and it increased the frictional force between the diamond particles and surface of the plated interlayer; hence, the diamond particles easily attached to the surface of the plated interlayer. Second, since diamond does not possess electrical conductivity, the electrical conductivity after a plated Cu interlayer is enhanced; the behavior of the electrode with solution interface was in the form of an electrical double layer. The plated Cu interlayer reduced the electrical double-layer impedance, thus increasing the actual current density and accelerating the reduction of Cu ions in solution at the cathode. At the same time, the accelerated reduction enhanced the diffusion of diamond particles and the adsorption action at the cathode. When parts of the ions adsorbed on the diamond particles were reduced, the particles were captured, and thus a composite deposition layer was formed with the matrix metal.

**Figure 4:**
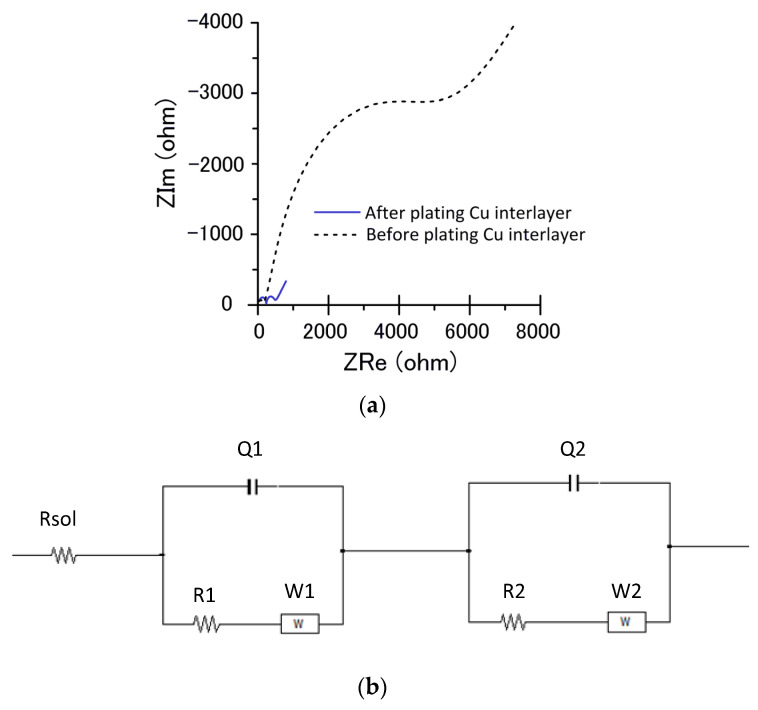
Impedance spectroscopy before and after a plated Cu interlayer and its equivalent circuit. (**a**) Impedance spectroscopy, and (**b**) equivalent circuit.

Electrochemical impedance spectroscopy analysis was performed on the specimens with a plated Cu interlayer and without one. Figure 4 shows an equivalent circuit for the electrode–electrolyte interface, which makes the error between the fitting value and the measured value less than 5%. Rsol is the solution resistance between the working electrode and the reference electrode. R1 and R2 are the faradaic charge transfer resistance. W1 and W2 are Warburg resistance. Q1 and Q2 are the equivalent electrical capacities. The impedance values after fitting are shown in Table 3. The magnitude of the electrical transfer impedance was determined by the diameter size of the semicircle in the impedance spectroscopy [33]. According to the plot, the electrical transfer impedance decreased after a plated Cu interlayer, suggesting that the transfer rate of electrons to the cathode improved after a plated Cu interlayer. Table 3 displays the electrochemical impedance values before and after a plated Cu interlayer. The significant reduction of the faradaic transfer resistance R2 from R1 also indicated the promotion of the Cu-plated specimen’s conductivity.

**Figure 5:**
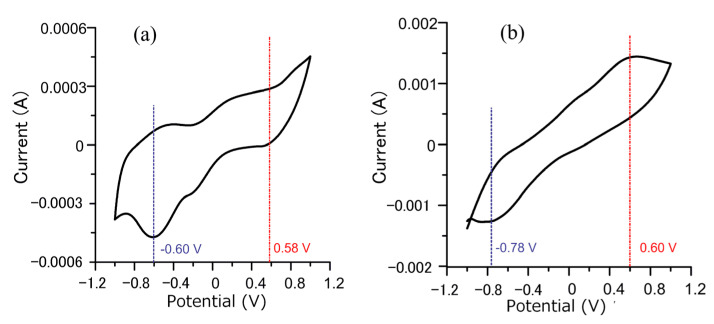
Cyclic voltammetry curves of the specimen (**a**) without Cu interlayer, and (**b**) with Cu interlayer.

Figure 5 shows the cyclic voltammetry curves before and after a plated Cu interlayer. Relative to the reference electrode, the reduction potential before a plated Cu interlayer was −0.6 V, and the oxidation potential was 0.58 V. After a plated Cu interlayer, the oxidation potential remained to around 0.6 V, and the reduction potential became more negative, at −0.78 V. The range between reduction and oxidation potentials became broader, and the reduction potential increased more, indicating enhancement of the reduction property. Furthermore, the current of the redox reaction on the surface of the plated Cu interlayer increased a lot, which means the electrodeposition rate increased significantly. As pointed out by the Guglielmi model theory, particle adsorption possesses an electrochemical property that depends on the electric field at the cathode [34]. The reaction was irreversible. According to the cyclic voltammetry curves, the increase in current by an order of magnitude with the plated Cu interlayer suggests that the redox reaction is enhanced, the relative current density was increased, and the reduction rate of copper ions was accelerated. The deposition rate of particles was related to the reduction rate of metal ions adsorbed on the particles [35]. Through the influence of the surrounding copper ions, the adsorption action of diamond particles at the cathode was enhanced, and the deposition rate was accelerated to increase the coating’s diamond particle content.

### 3.2. Morphology of Composite Coatings with Cu Interlayer and without Cu Interlayer

**Figure 6:**
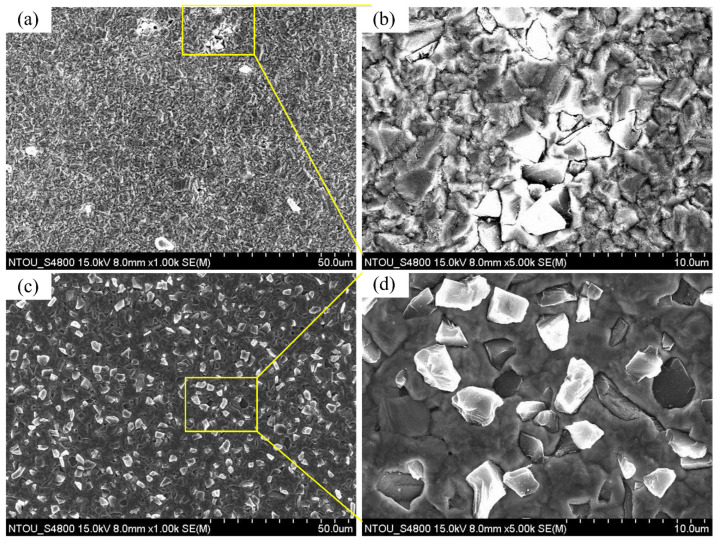
Surface morphologies of specimen A and specimen B: (**a**) Surface of specimen A under 1000× magnifications, (**b**) surface of specimen A under 5000× magnifications, (**c**) surface of specimen B under 1000× magnifications, and (**d**) surface of specimen B under 5000× magnifications.

Figure 6a shows the surface morphology of A0 under 1000× magnifications. There were very few diamond particles in the coating. The particle agglomerations induced very non-uniform diamond distribution in the coating. Figure 6b shows the surface morphology of the coating of specimen A under 5000× magnifications (diamond agglomeration zone). The overlap of diamond particles was observed, which resulted in surface irregularity of the coating. Figure 6c shows the surface morphology of the coating of specimen B under 1000× magnifications. The diamond distribution on the surface of the composite electrodeposition with a plated Cu interlayer was uniform and the dispersion of diamond particles was good. Morphological observation of specimen B under 5000× magnifications in Figure 6d shows that, as no defects such as cracks or pores were generated, the adhesion of diamond particles with surrounding copper should be good.

**Figure 7:**
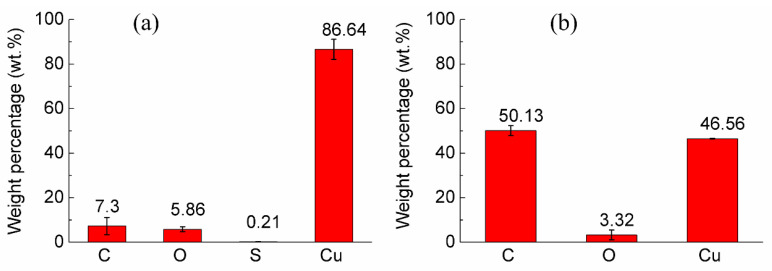
The weight percentages of coatings in each specimen: (**a**) Specimen A, and (**b**) specimen B.

The composition analysis of the coatings was conducted by the EDX at mapping mode on a 1 k image. Atomic percentages of specimen A and specimen B at three different positions were detected and shown in Figure 7a,b. The error bar is the standard deviation. The carbon weight percentage (i.e., diamond) of specimen A and specimen B was 7.30% ± 3.85% (*n* = 3) and 50.13% ± 2.27% (*n* = 3), where *p* < 0.0001. The diamond content of specimen B was significantly higher than that of specimen A. At the same time, the standard deviation of specimen B was smaller than that of specimen A, which indicated that the distribution uniformity of diamond particles in the coating of specimen B was better than that of specimen A. It was consistent with the uniform diamond distribution in the coating of specimen B and the clusters of diamond particles of specimen A in the former microscopic morphology analysis.

### 3.3. Characterization and Adhesion Strength of the Coatings

**Figure 8:**
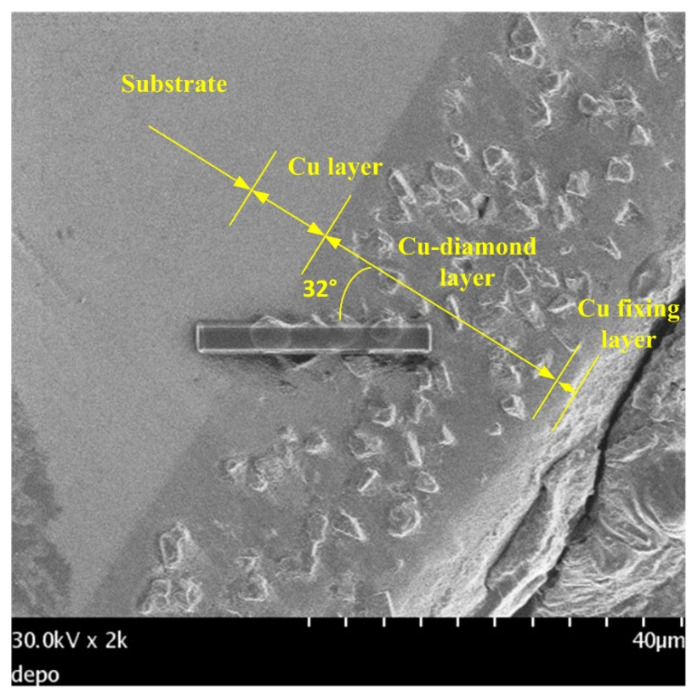
Cross-sectional view of the coating of specimen B.

**Figure 9:**
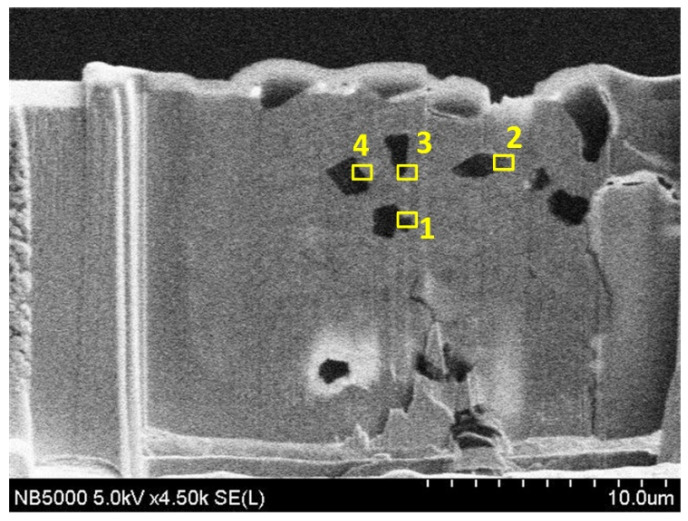
Cross section of specimen B after cutting by FIB and 1–4 representing different positions.

**Figure 10:**
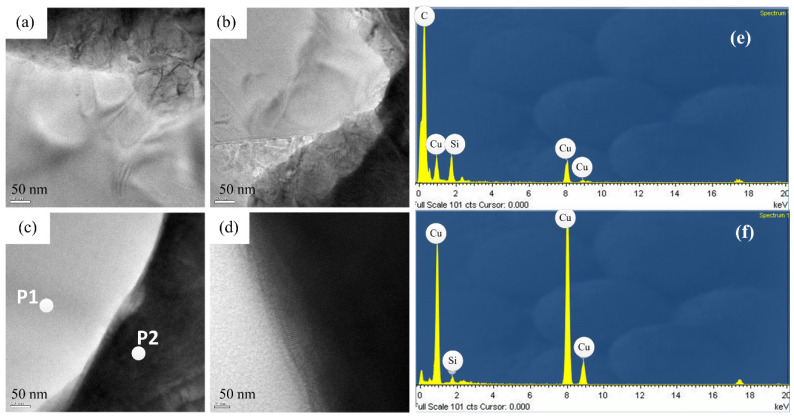
TEM micrographs of specimen B at different positions, (**a**) position 1, (**b**) position 2, (**c**) position 3, (**d**) position 4, (**e**) the element composition of P1, and (**f**) the element composition of P2.

Figure 8 shows a cross-sectional view of the coating of specimen B. The entire coating contained three layers of structure. The first layer was a plated Cu interlayer, the second layer was a composite electrodeposited copper–diamond layer, and the third layer was a reinforced Cu layer. The entire coating thickness was 32.650 ± 0.833 μm. Defects or voids were not observed at the interface between the Cu layer and the substrate [36,37]. Diamonds were uniformly distributed in the direction of the coating depth. Figure 9 shows the morphology of specimen B after being cut by FIB. The cutting direction was 32° with respect to the perpendicular direction to the coating’s surface. In the image, the yellow areas labeled 1, 2, 3, and 4 were analyzed using a TEM. In Figure 10a–d are the combined micrographs of diamond particles and matrix copper in the areas labeled as 1–4, respectively. According to the images, the diamonds were combined and closely packed with copper and featured no generated holes, gaps, or bubbles, illustrating that the diamond particles had good adhesion property with matrix copper, and the plated Cu interlayer was conducive to improve the interface adhesion [26].

The EDS element composition at the P1 and P2 positions are shown. There were Si and Cu elements at both positions, because the film was grounded in a copper grid, which contained Si and Cu components. In Figure 10e, the carbon was the major component, while in Figure 10f, the Cu was the major component, thereby it can be determined the component was diamond at P1 and Cu at P2.

**Figure 11:**
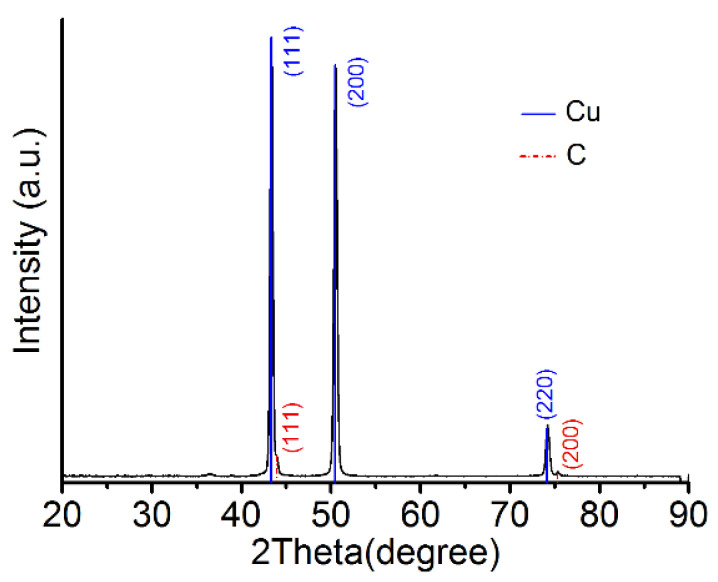
XRD spectra of the coating surface.

**Figure 12:**
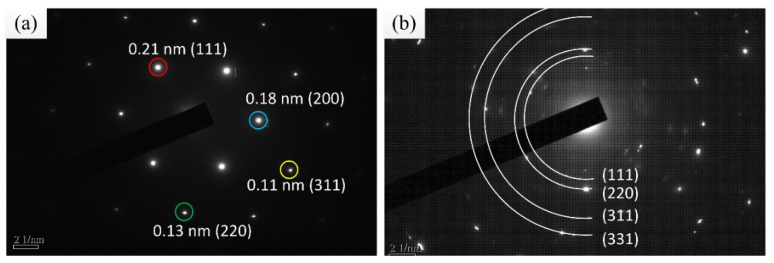
TEM diffraction pattern and corresponding Miller indices: (**a**) Cu, (**b**) diamond.

Using X-ray diffraction (XRD), Cu was found to crystallize in a face-centered cubic (fcc) lattice with a B1 (NaCl) structure (Cu PDF code: 00-004-0836), and the crystallization directions were (111), (200), and (220), in which (111) was the preferred direction. Diamond was found to be polycrystalline in an fcc lattice (PDF code is 03-065-0537), and the crystallization directions were (111), (200), and (311), with the preferred direction of diamond also being (111), as shown in Figure 11. The TEM diffraction pattern also confirmed the fcc structure of the Cu–diamond composite coating. The lattice parameter for the Cu is shown in Figure 12a, which was consistent with the XRD result, where diamond was found to be polycrystalline in an fcc lattice, with the lattice parameter for the diamond shown in Figure 12b, which was consistent with the XRD result, as shown in Figure 11.

### 3.4. Scratch Test Results

**Figure 13:**
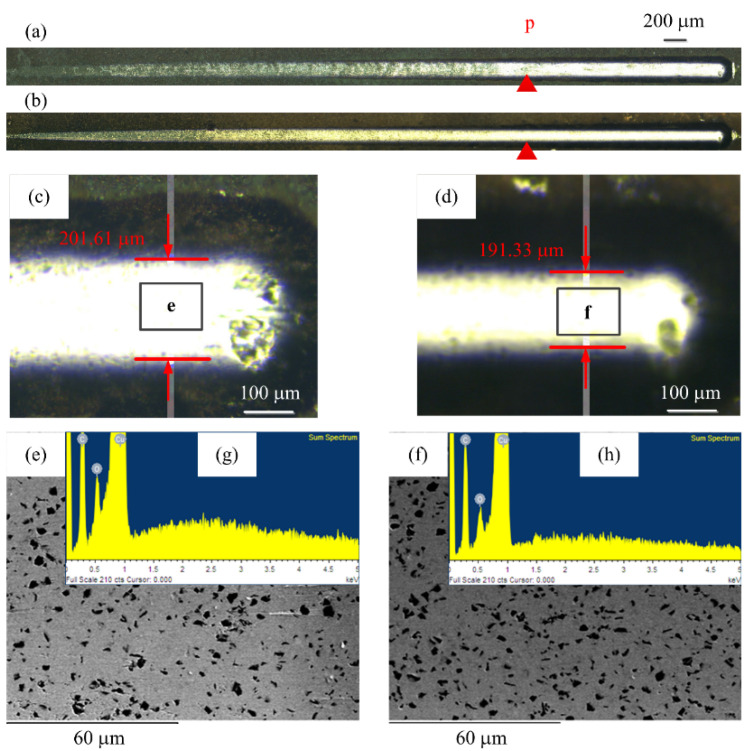
The scratch tracks of coatings: (**a**) Specimen A, (**b**) specimen B, (**c**) specimen A (end image), (**d**) specimen B (end image), (**e**) top morphology for (**c**), and (**f**) top morphology for (**d**).

The scratch test was carried out by implementing the Anton Paar Scratch Tester. Figure 13a,b shows the scratch morphology of specimen A and specimen B. Figure 13d shows the magnified end morphology for Figure 13a,b, respectively. The scratch width was 201.61 μm and 191.33 μm, respectively. They were affected by the diamond content and distribution of the coating. The scratch width of specimen B was smaller than that of specimen A, because the diamond content and distribution in the coating with the Cu interlayer was higher and more uniform than that of without the Cu interlayer, and it was more difficult to scratch in depth. The stylus penetrated through the coating at the P position. The surface morphologies at the end of the scratch for specimen A and specimen B are shown in Figure 13e,f. The carbon composition was investigated by EDX spectrum, as shown in Figure 13g,h, which verified the particles were diamond. Since the substrate possesses certain stiffness and is softer than the pure copper co-plated with diamonds, most diamond particles were removed, while the others were pressed into the copper substrate, which demonstrated that the adhesion between the multilayer coatings and substrate was strong. The press-in mechanism of the composite coatings is beneficial to prevent the diamond from being pulled off, and helps to prolong the service life of the coatings.

## 4. Conclusions

In this study, diamond-incorporated copper metal matrix composite layers were fabricated by using a three-step process on brass substrates. The adhesion and diamond content of the electrodeposited composite layers on the specimens with and without a plated Cu interlayer were analyzed. The results indicated that the composite coating with a Cu interlayer possessed significantly higher diamond content and more uniform distribution than that without one. Two major advantages of the Cu interlayer were identified. Firstly, the roughened surface, due to the plated Cu interlayer, provided numerous nucleation sites for the later electrodeposition and facilitated the mechanical interlock between the composite layers and the substrate. The enhanced adhesion of the coating was hereby achieved. Secondly, the lower surface impedance of the plated Cu interlayer increased the current density and accelerated the co-deposition of copper–diamond particles, which promoted the diamond content in the composite coating. As there were no defects such as cracks and voids in the coating, the excellent integration between the diamond particles and the copper metal matrix, and between the composite layers and the substrate, was verified. The end width of the scratch track on the composite coating with a Cu interlayer was smaller than that without one, which revealed the superior adhesion of the former one. The remaining diamond particles of both Cu-microdiamond coatings were pressed into the substrate along the scratch track, which indicated that the unique press-in mechanism could protect the diamond particles from being pulled off during the machining process.

## Figures and Tables

**Table 1:** Parameters of the electrolyte solution and the composite electrodeposition process.

Parameters	Values
CuSO_4_·5H_2_O (g/L)	250
H_2_SO_4_ (M)	0.5
Diamond’s particle size (μm)	2–4
Concentration of Diamond in electrolyte (g/L)	10
Current density (A/dm^2^)	5
Stirring speed (rpm)	200
Spacing between anode and cathode (cm)	5
Temperature (°C)	25

**Table 2:** Process durations for the specimens.

Specimen’s Code	Cu Interlayer (min)	Composite Electrodeposition (min)	Cu Fixing Layer (min)
A	/	27	3
B	5	22	

**Table 3:** Electrochemical impedance values before and after a plated Cu interlayer.

Specimen’s Condition	R1	R2	Q1	Q2	Rsol	W1	W2
Before a plated Cu interlayer	133.6	3600	2.88× 10^−8^	3.475× 10^−^^5^	11.8	2.857× 10^−4^	18.56× 10^−4^
After a plated Cu interlayer	226.8	216	1.139× 10^−5^	2.11× 10^−8^	22.92	26.77× 10^−4^	3.892× 10^−4^

## Data Availability

Data sharing not applicable.
